# Unbecoming a Gentleman

**Published:** 1864-04

**Authors:** J. W. Hunt

**Affiliations:** Recording Secretary


UNBECOMING A GENTLEMAN. We are requested to publish the following:—At a meeting of the District Medical Society for the county of Hudson, N. J., held February 15th, the following resolution was unanimously adopted:

WHEREAS, J. E. Quidor has been dismissed the service of the United States, "for conduct unbecoming a gentleman."

*Resolved*, That his name be erased, from the roll of membership, and the same be published in the *Medical and Surgical Reporter*, Philadeiphia, *New York Medical Times*, and *American Standard*, Jersey City.

J. W. HUNT, M. D.,
*Recording Secretary.*

We trust, that in cases where medical men in the army, disgrace their profession, by unbecoming conduct, they will uniformly be promptly dealt with by medical societies to which they may belong. In this respect the District Medical Society for the county of Hudson has set an example worthy of imitation. It is of the utmost importance that our profession be kept pure, and our medical societies owe it to themselves and to the public to deprive unworthy members whether in military or civil life, of the ægis of their protection. A drunken, ill-mannered, dishonest, boasting, ignorant medical man, should find no place within the sacred portals of any medical society, but should be "put out" immediately, and kept out until he "brings forth fruit meet for repentance."

*Medical and Surgical Reporter.*

